# Targeting and exploitation of tumor-associated neutrophils to enhance immunotherapy and drug delivery for cancer treatment

**DOI:** 10.20892/j.issn.2095-3941.2019.0372

**Published:** 2020-02-15

**Authors:** Yuting Zhang, Liu Guoqiang, Miaomiao Sun, Xin Lu

**Affiliations:** ^1^Department of Biological Sciences, Boler-Parseghian Center for Rare and Neglected Diseases, Harper Cancer Research Institute, University of Notre Dame, Notre Dame, IN 46556, USA; ^2^Integrated Biomedical Sciences Graduate Program, University of Notre Dame, Notre Dame, IN 46556, USA; ^3^Tumor Microenvironment and Metastasis Program, Indiana University Melvin and Bren Simon Cancer Center, Indianapolis, IN 46202, USA

**Keywords:** Tumor-associated neutrophil, polymorphonuclear myeloid-derived suppressor cell, immunosuppression, cancer immunotherapy, nanoparticle drug delivery

## Abstract

Neutrophils, the most abundant leukocytes in human blood, are essential fighter immune cells against microbial infection. Based on the finding that neutrophils can either restrict or promote cancer progression, tumor-associated neutrophils (TAN) are classified into anti-tumor N1 and pro-tumor N2 subsets. One of the major mechanisms underlying the tumor-promoting function of N2-TANs is suppression of adaptive immune cells, in particular, cytotoxic T lymphocytes. Currently, no established methodologies are available that can unequivocally distinguish immunosuppressive TANs and granulocytic/polymorphonuclear myeloid-derived suppressor cells (G/PMN-MDSC). In view of the critical role of PMN-MDSCs in immune evasion and resistance to cancer immunotherapy, as established from data obtained with diverse cancer models, therapeutic strategies targeting these cells have been actively developed to enhance the efficacy of immunotherapy. Here, we have reviewed the available literature on strategies targeting PMN-MDSCs and summarized the findings into four categories: (1) depletion of existing PMN-MDSCs, (2) blockade of the development of PMN-MDSCs, (3) blockade of PMN-MDSC recruitment, (4) inhibition of immunosuppressive function. Owing to their high mobility to inflamed organs and ability to trespass the blood-brain barrier, neutrophils are outstanding candidate carriers in nanoparticle-based therapies. Another attractive application of neutrophils in cancer therapy is the use of neutrophil membrane-derived nanovesicles as a surrogate of extracellular vesicles for more efficient and scalable drug delivery. In the second part of the review, we have highlighted recent advances in the field of neutrophil-based cancer drug delivery. Overall, we believe that neutrophil-based therapeutics are a rapidly growing area of cancer therapy with significant potential benefits.

## Introduction

Neutrophils are the most abundant population of white blood cells and often the first responders to the site of infection. Neutrophils are activated to kill bacterial pathogens *via* a variety of mechanisms, including phagocytosis, degranulation of antimicrobial factors, and release of neutrophil extracellular traps (NETs)^1–3^. Neutrophil differentiation and phenotype are profoundly influenced in multiple ways by solid tumors. Considerable evidence obtained from experimental tumor models and cancer patients supports morphological and functional heterogeneity of tumor-associated neutrophils (TANs)^4–9^. In a dichotomy similar to M1/M2 macrophages, TANs have been classified as tumor-inhibitory N1 and tumor-promoting N2 based on their functional differences^10^. Characteristic nuclear morphologies are potentially associated with N1-polarized and N2-polarized TANs. For instance, N1 neutrophils have hypersegmented nuclei whereas N2 neutrophils have banded or ring-like nuclei^10^. Recently, lectin-type oxidized LDL receptor-1 (LOX1)^11^ and fatty acid transport protein 2 (FATP2)^12^ were identified as distinguishing factors for the two populations of neutrophils in cancer patients, representing a significant advance in the classification of functionally distinct neutrophils based on molecular markers. Despite being a convenient model to describe TANs, N1/N2 classification is an oversimplification of neutrophil heterogeneity. Further molecular characterization of TANs based on single cell gene expression analysis is warranted to define the spectrum of neutrophil polarization within the tumor microenvironment.

The distinction between TANs and granulocytic/polymorphonuclear myeloid-derived suppressor cells (G/PMN-MDSCs) is a controversial issue^7^. Both share a cellular lineage origin and several morphological and phenotypic features, and currently, no surface markers are available that can be readily used for distinguishing the two cell types. By definition, the difference is that PMN-MDSCs potently suppress T cells whereas TANs may or may not possess immunosuppressive activity^13–15^. Considering N1/N2 classification, PMN-MDSCs and N2 neutrophils appear equivalent or essentially the same population. We believe that PMN-MDSC still presents a valid and helpful definition to describe a specialized neutrophil state that promotes tumor progression mainly through dampening adaptive immunity. Therefore, in this review, we will refer to immunosuppressive neutrophils and PMN-MDSCs interchangeably to indicate the population of TANs with immunosuppressive activity.

In view of the dominant negative regulatory role of PMN-MDSCs in anti-tumor immunity in many experimental cancer models, significant research has focused on understanding and targeting this cell population as a means of sensitizing tumors to current immunotherapies. Meanwhile, a separate yet related line of research has exploited the strong chemotactic property of neutrophils to deliver tumor-killing drugs. In the current review, we focus on two aspects, specifically, immunosuppressive neutrophil targeting and neutrophil-based drug delivery. For details on functions and mechanisms of TANs and PMN-MDSCs, readers may refer to a number of recent outstanding reviews^1,7,16,17^.

## Targeting of immunosuppressive neutrophils

Immunotherapy has revolutionized cancer treatment and become a new pillar of cancer therapy. Among the various immunotherapy modalities developed to date, immune checkpoint blockade (ICB) *via* antibodies targeting immune checkpoint molecules, such as cytotoxic T-lymphocyte-associated antigen 4 (CTLA4) and programmed death 1/programmed death ligand 1 (PD-1/PD-L1), has made the most significant impact^18,19^. However, the efficacy of immunotherapy is limited to a subset of patients and specific cancer types, mainly due to *de novo* or acquired resistance to immunotherapy^20,21^, and driven by tumor-intrinsic or tumor-extrinsic factors^22,23^. Among the resistance mechanisms, the impact of tumor-infiltrating PMN-MDSCs is one of the central barriers that impairs cytotoxic T lymphocytes (CTL) activity within the tumor bed. Thus, strategies targeting PMN-MDSCs, in combination with immunotherapy, present a promising approach to overcome resistance to immunotherapy. The currently available methods of PMN-MDSC targeting are classified as: (1) depletion of existing PMN-MDSCs, (2) blockade of the development of PMN-MDSCs, (3) blockade of PMN-MDSC recruitment, and (4) inhibition of immunosuppressive function.

### Depletion of existing PMN-MDSCs

**Figure 1:**
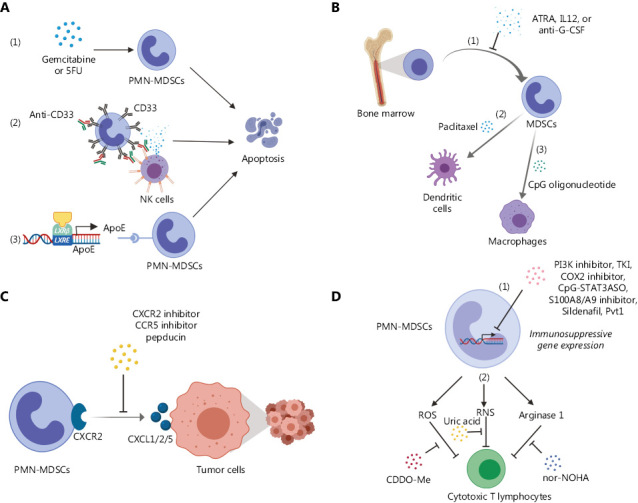
Different strategies of targeting immunosuppressive neutrophils. (A) Depletion of existing PMN-MDSCs: (1) Chemotherapeutic drugs, gemcitabine and 5-fluorouracil (5FU), directly induce apoptosis; (2) The anti-CD33 antibody, AMV564, induces NK cell-mediated antibody-dependent cellular cytotoxicity (ADCC); (3) LXRβ agonists activate ApoE and induce apoptosis. (B) Blockade of the development of PMN-MDSCs: (1) The differentiation process from myeloid progenitor cells in bone marrow to MDSCs is blocked by ATRA, IL-12, or anti-G-CSF antibody; (2) Ultra-low doses of paclitaxel induce differentiation of MDSCs into non-immunosuppressive dendritic cells; (3) CpG oligonucleotide induces MDSC to differentiate into non-immunosuppressive macrophages. (C) Blockade of PMN-MDSC recruitment: Delivery of PMN-MDSCs into the tumor microenvironment *via* chemotaxis is effectively blocked by CXCR2 inhibitors (such as SB225002 and SX-682), neutralizing antibody or peptide (pepducin), mCCR5-Ig or other agents. (D) Inhibition of PMN-MDSC immunosuppressive potential: (1) Immunosuppressive gene expression programs in PMN-MDSCs are blocked by PI3K inhibitors, RTK inhibitors, COX2 inhibitors, CpG-STAT3ASO, S100A8/A9 inhibitors, the PDE5 inhibitor sildenafil or long noncoding RNA Pvt1; (2) Immunosuppressive products from PMN-MDSCs, such as ROS, RNS and Arginase 1, are neutralized by nor-NOHA, uric acid, and bardoxolone methyl. The figure was generated with BioRender.com.

Chemotherapeutic drugs are reported to adversely affect MDSC viability. One of the typical side-effects of gemcitabine, a chemotherapeutic agent for many cancer types, including pancreatic cancer, is myelotoxicity. A study by Eriksson et al.^24^ demonstrated that gemcitabine is capable of reducing circulating MDSC and TGFβ-1 levels and elevating the effector T cell: Treg ratio in human pancreatic adenocarcinoma. The group showed a significant decrease in granulocytic but not monocytic MDSCs in the peripheral blood of patients with pancreatic adenocarcinoma eight days after gemcitabine treatment, supporting the utility of gemcitabine in depleting PMN-MDSCs and enhancing immunotherapy. Another chemotherapeutic agent, 5-fluorouracil (5FU), causes cell death by preventing synthesis of DNA and RNA^25^. Vincent and co-workers showed that 5FU induced a decrease in the number of MDSCs in the tumor microenvironment *via* triggering apoptosis and promoting IFN-γ production by tumor-infiltrated T cells to enhance antitumor immunity in a mouse EL4 cancer model^26^. Moreover, 5FU showed no preference for killing monocytic over granulocytic MDSCs and exerted no significant effects on T, natural killer, dendritic or B cells. Therefore, both gemcitabine and 5FU are considered promising agents to eliminate PMN-MDSCs in cancer patients. The antitumor effects of these drugs may be mediated, at least in part, by their selective toxicity against PMN-MDSCs [**Figure 1A(1)**].

Targeting of surface markers presents an attractive depletion approach. In mouse models, anti-Gr1 or anti-Ly6G antibody is often used to deplete PMN-MDSCs. However, Gr1 or Ly6G (part of the Gr1 antigen) is absent in humans, restricting this approach to experimental models. In cancer patients, CD33^+^ is used in combination with other markers, such as HLA-DR^−^ CD15^+^, to identify PMN-MDSCs. Therefore, antibodies and their derivatives targeting CD33 are putative candidate agents for PMN-MDSC depletion. AMV564 is a novel CD33/CD3 tetravalent bispecific antibody currently in early phase clinical trials for relapsed or refractory acute myeloid leukemia (AML) (NCT03144245). Cheng and co-workers^27^ reported that AMV564 treatment efficiently decreased the number of MDSCs and increased the CD4^+^ and CD8^+^ T cell numbers in primary bone marrow microenvironments from patients with myelodysplastic syndromes. The CD33-targeted antibody drug conjugate, gemtuzumab ozogamicin (GO), was reapproved in 2017 for treatment of AML^28^. To improve treatment options, it is important to determine whether agents, such as AMV564 and GO, demonstrate anti-MDSC activity in patients with solid tumors and show combinatorial synergy with immunotherapy [**Figure 1A(2)**].

Novel strategies to stimulate apoptosis of MDSCs are under development. For example, Tavazoie et al.^29^ showed that apolipoprotein E (ApoE), which is transcriptionally activated by liver X nuclear receptor (LXR), reduces the survival levels of both tumoral and circulating granulocytic and monocytic MDSCs by triggering apoptosis in mouse and human cancer models [**Figure 1A(3)**]. LXRβ agonists, such as GW3965 and RGX-104, induced depletion of immunosuppressive MDSCs (both granulocytic and monocytic) and impairment of tumors. Through depletion of MDSCs, LXRβ agonists augmented the efficacy of anti-PD-1 immunotherapy in melanoma and lung cancer models. Currently, the efficacy of RGX-104 as monotherapy and in combination with ICB immunotherapeutics, such as Nivolumab or Ipilimumab, is being evaluated in a phase I clinical trial for patients with malignant solid tumors and lymphoma (NCT02922764).

Taken together, the literature suggests that PMN-MDSCs can be efficiently depleted using chemotherapeutic or biological agents and targeted therapeutics. When combined with immunotherapeutic strategies, these treatments significantly enhance the effectiveness of immunotherapy and prolong survival of cancer patients.

### Blockade of the development of PMN-MDSCs

An alternative strategy to reduce PMN-MDSC number is to redirect their developmental pathway, either by inhibiting the conversion of immature myeloid cells into PMN-MDSCs or inducing differentiation of PMN-MDSCs into other mature myeloid cells that possess limited immunosuppressive activity. PMN-MDSC formation is induced by tumor-derived cytokines, such as granulocyte macrophage-colony stimulating factor (GM-CSF), granulocyte colony stimulating factor (G-CSF), and vascular endothelial growth factor (VEGF)^30^. These cytokines are capable of promoting accumulation of MDSCs originating from bone marrow and blocking their differentiation into mature myeloid cells, such as granulocytes, dendritic cells, and macrophages^31,32^. Thus, inhibition of these growth factors, their receptors or implicated downstream pathways may impair the PMN-MDSC population. For example, reduction in the number and activity of PMN-MDSCs by all-trans retinoic acid (ATRA)^33^, IL-12^34^, and anti-G-CSF antibody^35^ have been reported [**Figure 1B(1)**].

Theoretically, normalizing aberrant differentiation of MDSCs back into mature myeloid cells with diminished immunosuppressive and enhanced antitumor activities presents an ideal strategy with little anticipated toxicity. A number of studies support this possibility. Paclitaxel is a well-known chemotherapy drug for several cancer types. Interestingly, Michels et al.^36^ demonstrated that ultra-low doses of paclitaxel, which neither increased MDSC apoptosis nor blocked MDSC generation, could stimulate differentiation of tumor-infiltrating MDSCs into mature dendritic cells in a TLR4-independent manner in a murine melanoma model [**Figure 1B(2)**]. Immunostimulatory CpG oligonucleotides delivered *via* intratumoral injection reduced the immunosuppressive activity of MDSCs in the tumor bed by stimulating their differentiation into macrophages with tumoricidal capability^37^ [**Figure 1B(3)**].

Overall, blockade of the PMN-MDSC development, either by blocking their generation or inducing differentiation into non-suppressive myeloid cells, is an efficient strategy to abolish immunosuppression induced by PMN-MDSCs.

### Blockade of PMN-MDSC recruitment

PMN-MDSCs are recruited *via* specific chemotactic pathways. Several chemokine signaling pathways are involved in PMN-MDSC recruitment to the tumor microenvironment. Inhibitors of chemokine receptors are therefore promising candidate agents that may aid in preventing PMN-MDSCs from entering the tumor bed and shaping the immunosuppressive microenvironment (**Figure 1C**). Earlier studies using CXCR2 inhibitors (SB225002 and SX-682), a neutralizing antibody and peptide (pepducin) confirmed CXCR2 signaling as the central pathway for recruitment of PMN-MDSCs. Notably, CXCR2 blockade inhibited tumor progression in various tumor models^38–41^ and restored the sensitivity of highly refractory tumor models to immunotherapy^41–43^, a testimony for a key role of PMN-MDSCs in immunosuppression and resistance to cancer immunotherapy. A fusion protein, mCCR5-Ig, was generated to hinder PMN-MDSC accumulation *via* blocking CCR5 signaling in melanoma models^44,45^. In animal models of breast cancer metastasis, blocking the γδT cell/IL-17/neutrophil axis by neutralization of IL17 or G-CSF prevented neutrophil accumulation and downregulated the T cell-suppressive phenotype^46^. Ongoing clinical trials are underway to determine whether blocking PMN-MDSC recruitment can effectively enhance cancer immunotherapy. For example, NCT03161431 is an ongoing phase I clinical trial evaluating a combination of the CXCR1/2 inhibitor, SX-682, and ICB antibody, pembrolizumab, in patients with metastatic melanoma.

Based on the above findings, it is reasonable to suggest that blocking recruitment of PMN-MDSCs presents an effective prophylactic approach to inhibit the immunosuppressive activity of the tumor microenvironment, which may improve the efficacy of immunotherapy.

### Inhibition of the immunosuppressive function of PMN-MDSCs

Downregulation of the immunosuppressive function of PMN-MDSCs can be achieved by either silencing the signaling that controls immunosuppressive activity or diminishing immunosuppressive products secreted by PMN-MDSCs. Such treatments may help reprogram the tumor microenvironment to have less hostility toward CTLs and improve the chances of success of cancer immunotherapy.

Various strategies have been developed to dampen the immunosuppressive activity of PMN-MDSCs [**Figure 1D(1)**]. Patnaik et al.^47^ observed that inhibiting receptor tyrosine kinase signaling with Cabozantinib in *pten*^-/-^ *p53*^-/-^ mouse prostate tumor models promoted infiltration of neutrophils with antitumor activities into tumors, attenuating cancer progression. In this context, CXCL12 and HMGB1 are two tumor-secreted neutrophil chemotactic factors that recruit CXCR4-expressing neutrophils with antitumor properties. Another study by our group demonstrated that other than recruiting N1-like neutrophils, cabozantinib as well as the dual PI3K/mTOR inhibitor, dactolisib, significantly downregulated immunosuppressive genes, such as *Arg1*, *Ncf1* and *Ncf4*, in tumor-infiltrating PMN-MDSCs through targeting PI3K signaling^43^. By suppressing PMN-MDSCs, these drugs synergized with ICB to eradicate both primary and metastatic prostate tumors in the *PB-Cre*^+^ *Pten^L/L^ p53^L/L^ Smad4^L/L^* mouse model^43^. Isoform-selective PI3K inhibitors, such as the PI3Kγ inhibitor PI-549^48,49^, PI3Kβ inhibitor GSK2636771^50^, PI3Kδ/γ inhibitor IPI-145^51^ and (-)-4-O-(4-O-β-D-glucopyranosylcaffeoyl) quinic acid^52^ were shown to suppress tumor-promoting myeloid cells, including PMN-MDSCs, and enhance immunotherapy. Given the importance of STAT3 in MDSC expansion and activation^32^, silencing of STAT3 in PMN-MDSCs *via* an antisense oligonucleotide tethered to CpG oligonucleotide (CpG-STAT3ASO) reduced circulating PMN-MDSCs and promoted the CTL to Treg ratio in prostate cancer models^53^. Tumor-derived COX2-PGE2 signaling plays a key role in promotion of PMN-MDSCs in both animal models and human PBMCs, and COX2 inhibitors, such as celecoxib and SC-236, have been shown to diminish the number and activity of PMN-MDSCs, in part, through inhibition of STAT3 in myeloid cells^54–60^. S100A8/A9 is a heterodimeric pro-inflammatory mediator involved in both chronic and acute inflammation through activation of TLR4- or RAGE-mediated inflammatory pathways^61^. In gastric cancer patients, PMN-MDSCs accumulating in the tumor bed secreted higher levels of S100A8/A9. Inhibition of S100A8/A9 and its receptor (RAGE) impaired the ability of PMN-MDSCs to suppress T cell proliferation and IFN-γ production^62^. The effects of S100A8/A9 on blood, splenic and tumoral MDSCs were also effectively abolished by a peptide-Fc fusion protein (peptibody), which outperformed anti-Gr1 antibody and completely depleted both granulocytic and monocytic MDSCs in lymphoid organs and tumors in mice without affecting pro-inflammatory immune cell types, such as dendritic cells^63^. Inhibition of phosphodiesterase-5 (PDE5) by sildenafil (Viagra, used to treat erectile dysfunction) induced downregulation of arginase 1 and nitric oxide synthase-2, thereby reducing activity of intratumoral MDSCs^64,65^. Surprisingly, a long noncoding RNA, Pvt1, was shown to regulate the immunosuppressive activity of PMN-MDSCs in the tumor microenvironment, and siRNA-mediated silencing of Pvt1 inhibited intratumoral PMN-MDSC activity in the Lewis lung carcinoma mouse model^66^ [**Figure 1D(1)**].

The anti-inflammatory factors (enzymes, cytokines, and reactive chemicals) produced by PMN-MDSCs additionally present promising targets. Treatments that act by inhibiting these factors are considered the last line of defense against the effects of PMN-MDSCs [**Figure 1D(2)**]. Reactive oxygen species (ROS) and reactive nitrogen species (RNS) are the main weapons used by PMN-MDSCs to cause tolerance or death of CTLs^67–71^ and tumorigenic mutations in epithelial cells^72^. Another major mechanism used by PMN-MDSCs to suppress T cells is production of Arginase-1 that leads to diminished L-arginine levels, affecting T cell function^73,74^. Arginase-1 remains in the inactive form within granules of neutrophils and becomes activated when neutrophils release their granule content^75,76^. The agents shown to neutralize or inhibit the production of these molecules include N-hydroxy-nor-l-arginine (nor-NOHA)^67^, uric acid^70,77^, and bardoxolone methyl^78^. A recent study by our group demonstrated that RNS from intratumoral PMN-MDSCs promoted nitration of Tyr394 of lymphocyte-specific protein tyrosine kinase (LCK) in CTLs and abolished T cell receptor signaling through disruption of the phosphorylation cascade^77^. Neutralizing RNS with uric acid was a satisfactory strategy that negated the effects of PMN-MDSCs and greatly promoted the efficacy of immunotherapy in both prostate and lung cancer models. However, the optimal approach to neutralize PMN-MDSC-derived RNS in cancer patients remains to be established.

A particularly interesting strategy to target N2 neutrophils is through blocking neutrophil extracellular traps (NET). NETs are composed of DNA fibers with granule-derived antimicrobial proteins (such as neutrophil elastase, cathepsin G, and MMP-9) secreted by neutrophils through a process known as NETosis^8^. NETs can immobilize and neutralize pathogens including fungi, bacteria, and viruses^79^. Recently, NETs have been shown to assist metastasis by trapping circulating tumor cells (CTCs), promoting cancer cell invasion, and awakening cancer cells from dormancy^80–83^. Strategies to inhibit NETosis or remove existing NETs could therefore present an effective way to diminish metastasis. The agents exerting anti-NETosis effects in cancer models include DNase I-coated nanoparticles that digest NETs^80^, antibodies against NET-remodeled laminin^81^, heparin that sequesters histone from NETs with its negative charges^83,84^, and inhibitors of protein-arginine deiminase 4 (PAD4), an enzyme required for NET formation^85^. Use of these inhibitors should be carefully evaluated in patients, since inhibition of NETs may also compromise their important functions in innate immunity.

### Challenges in selective targeting of immunosuppressive neutrophils

We have summarized the four main strategies employed to target PMN-MDSCs, including depletion using apoptosis-induced agents, blockade of the development, blockade of recruitment from the bone marrow, and elimination of immunosuppressive signaling and associated factors. Several important challenges and issues remain in developing selective therapeutics to target this population of neutrophils. First, the methodology used to define PMN-MDSCs in experimental models and clinical samples makes evaluation cumbersome. Due to the lack of definitive markers to distinguish PMN-MDSCs from other types of neutrophils, isolated intratumoral PMN-MDSCs must be subjected to a T cell co-culture assay to evaluate whether the therapeutic agent can diminish immunosuppressive activity. This method is feasible for experimental models but difficult to implement in clinical trials. Second, the signaling mechanisms used by PMN-MDSCs to suppress T cells are often hyperactive in other cancer cells or M2-polarized macrophages in the tumor microenvironment. Therefore, it is often difficult to attribute the therapeutic effect solely to PMN-MDSCs. Third, due to the complexity and redundancy of immunosuppressive mechanisms, blocking one mechanism may be compensated by other mechanisms, leaving T cells still suppressed. Therefore, blockage of PMN-MDSC recruitment may be a favorable strategy because without continuous recruitment from the bone marrow, tumors are expected to exhaust their supply of infiltrating PMN-MDSCs rapidly and become susceptible to killing by CTLs activated by immunotherapy. Fourth, most of the existing knowledge on PMN-MDSCs has been obtained from mouse models. However, the differences between human and mouse immune systems, especially in the context of neutrophils, and their roles in cancer and immunotherapy make translation of findings from mouse models to clinical therapy a long and difficult process. To address these challenges, future research should focus on identifying biomarkers highly specific for PMN-MDSCs and developing more selective therapeutics (antibody-drug conjugate or bispecific antibody based on highly selective PMN-MDSC surface markers). New humanized mouse models incorporating PMN-MDSCs, autologous cancer cells and T cells of human origin should be developed as a tool for elucidation of PMN-MDSC biology and therapeutics.

## Neutrophil-based drug delivery

As the most abundant immune cells in the circulation, neutrophils play an essential role in immune responses to tissue damage or infection. Upon inflammation, neutrophils are the first immunocytes recruited to the inflammation site^3^. Owing to high responsiveness and mobility, neutrophils present an outstanding candidate cellular carrier for various antitumor reagents^86^. So far, two main strategies have been employed: (1) use of live neutrophils and (2) use of neutrophil membrane-derived nanovesicles as drug delivery tools.

### Neutrophils as a drug delivery carrier

Targeting of solid tumors with nanoparticles (NPs) loaded with antitumor drugs is an attractive therapeutic option. However, the vast majority of administered NPs are not effectively delivered to solid tumors^87^. Limited NP-based delivery strategies have been successful in the clinic and solving the NP delivery problem should accelerate clinical translation of nanomedicine. The remarkable ability of neutrophils to migrate from the circulation to various tissues supports their utility as promising drug carriers to enhance the therapeutic effect^88–91^. Recently, Wang et al.^92^ showed that activated but not resting neutrophils or monocytes were able to take up drug-loaded albumin NPs in a Fcγ receptor-dependent manner. Neutrophils treated with albumin NPs loaded with Syk inhibitor, piceatannol, lost “outside-in” β2 integrin signaling activity and detached from the inflamed vasculature in vascular inflammation and lung injury models. This capability of activated neutrophils to take up NPs is potentially exploitable for cancer treatment.

**Figure 2:**
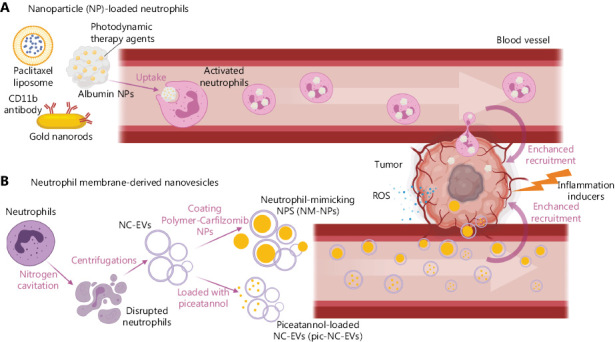
Neutrophil-based drug delivery. The inflammatory tumor microenvironment exacerbated by tumor-recognizing therapeutic antibodies, photosensitization or surgery enhances the recruitment of drug/NP-loaded neutrophils or neutrophil membrane-derived nanovesicles, which is the most essential prerequisite for neutrophil-based drug delivery. (A) NP-loaded neutrophils. Neutrophils carry therapeutic liposomes (loaded *ex vivo*, details not shown), albumins or CD11b antibody-coated NPs (engulfed by circulating neutrophils) and travel into the tumor microenvironment to deliver the drugs following chemoattraction by tumor-emitted pro-inflammatory signals. The neutrophils penetrate the blood brain barrier (BBB) to exert their effects. (B) Neutrophil membrane-derived nanovesicles. Nanovesicles generated using neutrophil membranes are loaded with drugs (such as piceatannol) or form neutrophil-mimicking NPs (NM-NPs). The neutrophil-derived properties of nanovesicles allow homing to the tumor microenvironment for therapeutic delivery. The figure was generated with BioRender.com.

In two studies, Chu et al.^93,94^ demonstrated that neutrophil-mediated delivery of NPs across the blood vessel barrier into tumor tissues after induction of inflammation is a valid approach to precisely deliver therapeutic agents in tumor models. In both investigations, acute stimulation of inflammation, either *via* a monoclonal antibody or photosensitization, was required for neutrophil recruitment. Neutrophils delivered pyropheophorbide-a loaded NPs or anti-CD11b antibody-linked gold nanorods to the tumor bed and induced a tumoricidal effect (**Figure 2A**).

A particular advantage of using neutrophils as the vehicle for drug delivery is their ability to penetrate the blood brain barrier (BBB) and reach inflamed brain tumors owing to high migratory capacity^95,96^. Xue et al.^97^ reported that neutrophils carrying paclitaxel (PTX) liposomes could penetrate the brain and suppress recurrence of glioma in mice whose tumors had been resected surgically. The pro-inflammatory factors generated at the site of resection triggered massive infiltration of neutrophils. The high concentration of inflammatory signals in the brain further triggered release of liposomal PTX from neutrophils, facilitating delivery of PTX to residual tumor cells (**Figure 2A**). As a result, neutrophil-mediated delivery of drugs efficiently impeded relapse and improved survival.

In contrast to the delivery of non-toxic agents, such as photosensitizers or gold NPs in an *in situ* setting, loading neutrophils with toxic anti-cancer drugs *ex vivo*, such as paclitaxel, is expected to be a significant challenge, since neutrophils are highly sensitive and have a short lifetime *ex vivo*. Moreover, chemotherapeutic drugs may significantly impair neutrophil viability and functions, such as cell migration^98^. Nevertheless, Xue and colleagues^97^ showed that PTX liposomes exert negligible toxicity to neutrophils compared with free PTX, suggesting that *ex vivo* loading of neutrophils with carefully designed chemotherapeutic drug-containing NPs is feasible and loaded neutrophils may survive long enough after infusion to effectively target tumors and generate the desired therapeutic effect.

While still an emerging field of research, studies to date have revealed that the use of neutrophils as vesicles for drug delivery can improve the efficacy of immunotherapy and aid in resistance to tumor recurrence. With broader application of immunotherapy in cancer treatment, we expect neutrophil-mediated drug delivery to become a valuable complementary approach.

### Neutrophil membrane-derived nanovesicles for drug delivery

Lipid-based nanomaterials, such as liposomes, are extensively used as carriers for drug delivery due to several suitable physical and chemical properties. However, the exogenous nature of liposomes may elicit immunogenicity *in vivo*, leading to compromised therapeutic effects and elevated risk of systemic immune responses^99,100^.

Extracellular vesicles (EV) are lipid bilayer-delimited particles naturally released from cells that are loaded with proteins, lipids, nucleic acids and sometimes even organelles. These vesicles play important roles in intercellular communication under both physiological and pathological conditions. A variety of cell adhesion molecules are expressed on the EV surface, facilitating their utility as carriers for drug delivery^101^. However, clinical application of EVs is currently impeded by low yield, inefficient drug loading and difficulty in scalability^102^.

Gao et al.^102^ reported a strategy to generate EVs using nitrogen cavitation (NC-EVs) that instantly disrupts neutrophils to form nanosized membrane vesicles. NC-EVs are similar to naturally secreted EVs (NS-EVs) by neutrophils but include less nuclear acids and organelles. Due to the high yield of production (16 times higher than NS-EVs), NC-EVs are potentially applicable for clinical use. In the study, NC-EVs loaded with piceatannol (an anti-inflammatory drug) alleviated acute lung inflammation/injury and sepsis induced by lipopolysaccharide in mice (**Figure 2B**). The data suggest that nitrogen cavitation is feasible for efficient manufacture of neutrophil-membrane-derived EVs on a large scale. This approach may therefore be adapted for the efficient production of chemotherapy drug-loaded NC-EVs.

A nanosized neutrophil-mimicking drug delivery system (NM-NP) was developed by Kang and co-workers by coating neutrophil membranes on the surface of poly (lactic-co-glycolic acid) NPs without damaging membrane-associated proteins and binding affinities^103^. Compared with uncoated NPs, NM-NPs displayed enhanced cellular association under shear flow *in vitro* and higher capture efficiency of CTCs *in vivo*. For therapeutic purposes, NM-NPs loaded with carfilzomib, a second-generation proteasome inhibitor, exhibited impressive activity in depleting CTCs in the circulation and inhibiting the initiation and development of metastasis in animal models (**Figure 2B**). This exciting study reveals the potential of of NP-loaded neutrophil-derived vesicles in treating metastasis, a direction warranting further investigation.

## Conclusions and perspectives

We have reviewed two related topics: (1) how to target immunosuppressive neutrophils and (2) how to use neutrophils to battle cancer. For targeting immunosuppressive neutrophils, four types of strategies have been developed, including depletion, redirection of differentiation, blockage of recruitment, and functional inactivation of PMN-MDSCs. These approaches require a deeper mechanistic understanding of neutrophils in cancer development and immune evasion. Among the many challenges of neutrophil targeting, perhaps the most prominent is molecular discrimination of TANs, especially PMN-MDSCs, from the remaining neutrophil populations required for innate immunity against bacterial and fungal pathogens. We believe that technological advances, especially single cell profiling techniques, such as single cell RNA-seq and singe cell proteomics, will lead to greatly enhanced abilities to classify heterogeneous neutrophil populations, pinpoint specific protein markers in “bad” neutrophils and spare “good” neutrophils to avoid neutropenia as a major side-effect of neutrophil-targeted therapy.

Neutrophils are a double-edged sword in cancer development. Strategies should be developed not only to disrupt pro-tumor neutrophils but also reinstate anti-tumor neutrophils. Application of neutrophils and their membranes for better drug delivery and novel therapeutics has already generated impressive results in preclinical models and neutrophil-based therapeutics present a rapidly growing area of cancer therapy. With the accumulating research efforts dedicated to targeting tumor-associated immunosuppressive neutrophils and devising novel neutrophil-based therapies, we expect significantly higher numbers of cancer patients to benefit from immunotherapy and personalized targeted therapies in the next decade.
